# Performance of hybrid progeny formed between genetically modified herbicide-tolerant soybean and its wild ancestor

**DOI:** 10.1093/aobpla/plv121

**Published:** 2015-10-27

**Authors:** Zheng-Jun Guan, Peng-Fei Zhang, Wei Wei, Xiang-Cheng Mi, Ding-Ming Kang, Biao Liu

**Affiliations:** 1 State Key Laboratory of Vegetation and Environmental Change, Institute of Botany, Chinese Academy of Sciences, Beijing 100093, China; 2 Department of Life Sciences, Yuncheng University, Yuncheng, Shanxi 044000, China; 3 College of Agriculture and Biotechnology, China Agricultural University, Beijing 100193, China; 4 Nanjing Institute of Environmental Sciences, Ministry of Environmental Protection of China, Nanjing, Jiangsu 210042, China

**Keywords:** Genetically modified soybean, glyphosate resistant, hybridization, plant growth, wild soybean

## Abstract

Gene flow from genetically modified (GM) crops to wild relatives might affect the evolutionary dynamics of weedy populations and result in the persistence of escaped genes. To examine the effects of this gene flow, the growth of F_1_ hybrids that were formed by pollinating wild soybean (*Glycine soja*) with glyphosate-tolerant GM soybean (*G. max*) or its non-GM counterpart was examined in a greenhouse. The wild soybean was collected from two geographical populations in China. The performance of the wild soybean and the F_2_ hybrids was further explored in a field trial. Performance was measured by several vegetative and reproductive growth parameters, including the vegetative growth period, pod number, seed number, above-ground biomass and 100-seed weight. The pod setting percentage was very low in the hybrid plants. Genetically modified hybrid F_1_ plants had a significantly longer period of vegetative growth, higher biomass and lower 100-seed weight than the non-GM ones. The 100-seed weight of both F_1_ and F_2_ hybrids was significantly higher than that of wild soybean in both the greenhouse and the field trial. No difference in plant growth was found between GM and non-GM F_2_ hybrids in the field trial. The herbicide-resistant gene appeared not to adversely affect the growth of introgressed wild soybeans, suggesting that the escaped transgene could persist in nature in the absence of herbicide use.

## Abstract (precis)

Gene flow may occur between genetically modified (GM) crops and wild relatives and the fate of escaped transgenes depends on the performance of hybrids and the fitness of the transgene. Hybrids were formed by hand-crossing a GM strain of glyphosate-tolerant soybean and its non-GM counterpart with wild soybean and were assessed in this study. The hundred-seed weight of hybrids was significantly higher than that of wild soybean. However, no overall difference in plant growth was found between GM and non-GM hybrids. The results suggest that the herbicide-resistant transgene may not pose fitness costs and could persist in nature.

## Introduction

Genetically modified (GM) crops produced using modern biotechnology have developed tolerances to biotic and abiotic factors, including herbicide and/or insecticide resistance. The release of GM crops has raised concerns that gene introgression could occur from these crops to wild or weedy populations (Snow 2002; Lu and Snow 2005; Andow and Zwahlen 2006). Performance measurement of hybrids can predict the ecological consequences of transgene spread from GM crops to wild relatives (Stewart *et al*. 2003; Hails and Morley 2005). The probability of transgene introgression into populations of compatible relatives is highly dependent on the performance of the F_1_ hybrid and the subsequent generations (Lu and Snow 2005; Laughlin *et al*. 2009). The performance of a single plant or group, which may indicate the adaptive, competitive and invasive ability of the plant population as a whole, may be obtained by analysing the traits associated with growth and reproduction and by comparing different individuals or groups, such as GM F_1_ and non-GM F_1_, or hybrid progeny and wild parent (Snow *et al*. 1999; Allainguillaume *et al*. 2006; Cao *et al*. 2009; Wang *et al*. 2014).

Since the worldwide commercial release of a GM soybean (*Glycine max*) that is resistant to the herbicide glyphosate, scientists have been studying its potential environmental effects (e.g. Coghlan 1999; Lorraine-Colwill *et al*. 1999; Elmore *et al*. 2001; King *et al*. 2001; McPherson *et al*. 2003; Kremer *et al*. 2005; Mizuguti *et al*. 2009; Zobiole *et al*. 2011). The risk of herbicide-resistant gene introgression from GM soybeans to conventional soybean or wild populations has become a priority consideration in biosafety assessment in countries with valuable diverse wild soybean resources. For instance, research showed that the GM glyphosate-resistant soybean AG5601 may pose a risk of gene ﬂow via pollination, allowing transgene escape to conventional soybean in China (Huang *et al*. 2014).

The annual wild soybean (*G. soja*) is an important genetic pool for soybean breeding and serves a crucial role in cultivar development. Studies have been conducted to determine the hybridization rate and to establish a field isolation distance between *G. soja* and cultivated GM soybeans, in order to minimize the possibility of outcrossing or other deleterious effects on this precious resource (Yoshimura *et al*. 2006; Lü *et al*. 2009; Zhao and Zhang 2012). Most research data suggest that escape of the transgene from the GM soybeans has indeed occurred into wild populations; however, it is unknown whether the transgene can exist stably in wild soybeans and whether it can be passed down to progeny plants. Research on the transgene escape of GM soybeans has mainly focussed on the frequency of gene flow and factors that affect gene flow (Nakayama and Yamaguchi 2002; Kitamoto *et al*. 2012). No study has evaluated the potential consequences of crop-to-wild introgression in soybeans by measuring the performance of hybrids between wild soybean and cultivated GM soybean.

China is the origin of the annual wild soybean, and spontaneous hybridization has occurred between wild and cultivated soybeans (Wang *et al*. 2010). Numerous soybean seeds have been imported into China for industrial production from overseas, which has been the main site of production of glyphosate-resistant GM soybeans. Some seeds may leak during transportation and become a source of GM gene flow. In addition, glyphosate-resistant soybeans and many herbicide-resistant GM soybean lines have been developed and tested in the field in China (Zhao and Zhang 2012). Therefore, it is necessary to assess transgene escape and its consequences in nature. In the present study, wild soybean was pollinated with GM glyphosate-resistant soybean and its non-GM counterpart. Selfing of F_1_ was permitted to obtain F_2_ progeny. The performance of GM F_1_ and F_2_ hybrids was evaluated in the greenhouse and in the field, respectively. We aimed to predict the risk and consequences of gene flow from GM soybean to wild soybean and the potential persistence of the transgene in nature. These results will assist in the biosafety management of GM soybean and advance scientific research on risk assessments of GM crops.

## Methods

### Plant materials

Cultivated GM soybean tolerant to glyphosate (AG5601) expressing the bacterial 5-enolpyruvylshikimate-3-phosphate synthase (EPSPS) enzyme and the non-GM glyphosate-susceptible counterpart (SKN500) (Wu *et al*. 2007) were provided by Monsanto Company (Beijing, China) and were used as the pollen donor (male parent). Wild soybean (*G. soja*) was collected from two counties (Miyun and Pinggu) in the Beijing area, China, and provided by Dr Xiang-hua Li of the Institute of Crop Science of the Chinese Academy of Agricultural Sciences. Wild *G. soja* was chosen as the pollen recipient (female parent) (Table 1).

**Table 1.** Types and number of plants used in the experiments.

Plant type	Parents	Greenhouse expt. (2010)	Field expt. (2011)
Wild soybean ‘Miyun’		13	9
GM F_1_-Miyun	Miyun (♀) × AG5601 (GM)	13	—
Non-GM F_1_-Miyun	Miyun (♀) × SKN500 (non-GM)	9	—
GM F_2_-Miyun	Selfing of F_1_ [Miyun (♀) × AG5601 (GM)]	—	28
Non-GM F_2_-Miyun	Selfing of F_1_ [Miyun (♀) × SKN500 (non-GM)]	—	41
Wild soybean ‘Pinggu’		7	14
GM F_1_-Pinggu	Pinggu (♀) × AG5601 (GM)	8	—
Non-GM F_1_-Pinggu	Pinggu (♀) × SKN500 (non-GM)	—	—
GM F_2_-Pinggu	Selfing of F_1_ [Pinggu (♀) × AG5601 (GM)]	—	38
Non-GM F_2_-Pinggu	Selfing of F_1_ [Pinggu (♀) × SKN500 (non-GM)]	—	—

### Hybridization between wild soybean and GM and non-GM soybean

Wild Miyun and Pinggu soybean seeds were sown in the field in May 2010. Four healthy plants from each of the two geographic collections were chosen and the stamens were emasculated at the flowering stage. Two plants of each of the two geographic collections were hand-pollinated with pollen from 30 plants of GM soybean AG5601, while the other two plants were hand-pollinated with pollen from 30 plants of non-GM soybean SKN500. To ensure simultaneous ﬂowering periods of female and male parents for successful crosses, the soybeans were sown on three different dates, with intervals of 10 days between the planting dates. Therefore, there were four sets of crosses: Miyun × GM (AG5601), Miyun × non-GM (SKN500), Pinggu × GM (AG5601) and Pinggu × non-GM (SKN500). The pod setting percentage for all the sets ranged from 0 to 9 % (Table 2) and was highest in Miyun × GM (AG5601). The fertility rate of the four crossing sets was assumed to be very low. Pinggu × non-GM (SKN500) produced no seeds; thus, no further assessment of their hybrids was conducted. The mature hybrid seeds (F_1_) that resulted from the remaining three hybridizing combinations were hand-harvested, air-dried and stored separately. The crossings produced varied numbers of seeds (Table 2).

**Table 2.** Hybrids (F_1_) formed between two wild soybean accessions and two cultivated soybeans (GM and non-GM).

Pollen recipient (♀)	Pollen donor (♂)	No. of pollinated flowers	Rate of pod setting (%)	No. of harvested seeds	No. of tested seeds/seedlings	No. of identified hybrids	Hybridization rate (%)
Wild soybean-‘Miyun’	AG5601 (GM)	100	9	25	20	13	65
	SKN500 (non-GM)	100	6	16	9	9	100
Wild soybean-‘Pinggu’	AG5601 (GM)	100	6	18	12	8	67
	SKN500 (non-GM)	100	0	0			0

### Greenhouse and field experiments

In November 2010, hybrid plants were produced for the greenhouse trial. All plump seeds [20 F_1_ seeds of Miyun × GM (AG5601), 9 F_1_ seeds of Miyun × non-GM (SKN500) and 12 F_1_ seeds of Pinggu × GM (AG5601); Table 2] were soaked in petri dishes after seed coat cutting to generate full germination and then transplanted to Jeffy-7 peat pellets. Hybrid seedlings identified by polymerase chain reaction (PCR; see details below) were transplanted to pots in the greenhouse at the three-leaf and five-leaf growth stages (Table 2). In addition, 25 wild soybean seedlings (15 Miyun and 10 Pinggu plants) were all transplanted to generate enough controls of wild plants for assessing the performance of F_1_ plants in the greenhouse environment. Of these seedlings, only 13 Miyun and 7 Pinggu strong seedlings (Table 1) survived to produce seeds, which were included in the final analysis.

The harvested seeds of F_1_ plants in the greenhouse were labelled as GM or non-GM F_2_ using the geographical names of their maternal parents (Miyun or Pinggu) as suffixes. For plant performance evaluation, harvested wild soybeans and F_2_ seeds were sown in May 2011 in the experimental field of China Agricultural University in Beijing, China (40°08′N, 116°10′E). The seeds were sown in three blocks containing a total of 270 spots. Wild Miyun, wild Pinggu GM F_2_-Miyun, non-GM F_2_-Miyun and GM F_2_-Pinggu were distributed in 45, 45, 60, 60 and 60 spots at sowing. Those spots were placed 100 cm apart in a zigzag pattern along each row to avoid interaction between any two neighbour plants. Seeds of the five plant types were sown randomly among the spots (three seeds in each spot).

At the three-to-four leaf stage, only one GM-F_2_ plant carrying the EPSPS gene or one wild soybean plant was retained at each spot, according to PCR identification. Due to unexpected dry climate conditions and/or potential seed dormancy, especially in wild soybean, which possesses a hard seed coat (Sun *et al*. 2015), the five plant types were unequally represented in the field samples. We harvested 9 wild soybean Miyun plants, 14 wild soybean Pinggu plants, 28 GM F_2_-Miyun plants, 38 GM F_2_-Pinggu plants and 41 non-GM F_2_-Miyun plants (Table 1). The seeds harvested from F_2_ plants in the field were considered as F_3_ seeds.

The following plant growth data for each harvested plant were recorded: (i) the date of the first flower opening for each plant, in order to calculate the number of days from planting to ﬂowering, defined as the period of vegetative growth (after the flowering stage, the plants entered the reproductive growth period), (ii) above-ground dry biomass, (iii) number of pods per plant, (iv) number of seeds per plant and (v) 100-seed weight.

### Polymerase chain reaction detection of the EPSPS gene in hybrids

To verify the existence of the glyphosate-resistant gene in F_1_ and F_2_ plants, we employed PCR to test the three-to-four leaf stage of the F_1_ and F_2_ seedlings. Plant genomic DNA was extracted from leaves using a Miniprep kit (Tiangen Biochemical Technology Co., Ltd, Beijing, China). To amplify the 146-bp fragment of EPSPS gene in an F_1_ seedling, a pair of primers were used (forward primer sequence: 5′-GCAAACCTCTGGCCTTTCC-3′; reverse primer sequence: 5′-CTTGCCCGTATTGATGACGTC-3′) (Lü *et al*. 2003). Another pair of simple sequence repeat (SSR) primers specific to cultivated soybean was used to detect F_1_ hybrids produced between wild soybean and non-GM soybean (SKN500) (forward primer: 5′-GCGTGTGCAAAATGTTCATCATCT-3′; reverse primer: 5′-GGCACGAATCAACATCAAAACTTC-3′) (see Kuroda *et al*. 2006). Each PCR reaction was carried out in a 10-µL reaction volume. Each mixture contained 1 µL of 10× Taq buffer, 0.8 µL of dNTP mixture (2.5 mM each), 0.2 µL of each primer (10 µM), 0.2 µL of TaKaRa Taq DNA polymerase (2.5 U µL^−1^) [TaKaRa Biotechnology (Dalian) Co., Ltd] and ∼20 ng of genomic DNA. The PCR amplification was run on a Biometra thermocycler with the following thermocycle profile: 94 °C for 3 min for initial denaturation, followed by 35 cycles of 94 °C for 30 s, 55 °C for 30 s and 72 °C for 1 min, and terminated by a final extension at 72 °C for 5 min. Amplified DNA products were separated on 2 % agarose gels at 100 V for 1 h, in 1× tris-borate-ethylene diamine tetraacetic acid buffer, stained with ethidium bromide and visualized under a Bio-Rad transilluminator. The respective wild soybean and GM soybean samples were used as negative and positive controls for all tests.

### Statistical analysis

General linear model analysis was performed among various plant types using SPSS16.0 software. Tests for significance were conducted for five variables, including vegetative growth period, above-ground biomass, pod number, seed number and 100-seed weight per plant. According to the homogeneity of variances, the means of each variant were tested for multiple comparisons between different plant types either by Duncan's multiple range test or by Tamhane's multiple range test, followed by a Bonferroni correction (*α* = 0.05).

## Results

### Characteristics of F_1_ hybrid and F_2_ progeny

Hybrid seeds were intermediate in size between wild soybean and cultivated soybean. The successful GM hybrid had an EPSPS gene fragment of 146 bp **[see** **Supporting Information—Fig. S1****A and B]**, and the successful non-GM hybrid F_1_ detected by SSR–PCR had two bands [one the same as the wild female parent (100 bp), and the other the same as the male crop parent (300 bp)] **[see** **Supporting Information—Fig. S1****C]**. The presence of the transgene in the F_1_ seeds sets on wild soybean from two sets of hand-crosses by GM plants was <100 % [65 % in F_1_ hybrid of GM (AG5601) × Miyun and 67 % in F_1_ hybrid of GM (AG5601) × Pinggu] (Table 2). In contrast, the tested plants of non-GM (SKN500) × Miyun were all hybrids (100 %).

The segregation rate of the transgene in F_2_ progeny of wild soybeans from different geographical populations had different levels of deviation, although the limited sample size may have affected this result (Table 3). The segregation rate of GM (AG5601) × Pinggu for glyphosate resistance significantly deviated from 3 : 1 (χ^2^= 5.44, *P* = 0.014), and the transgene segregating rate of GM (AG5601) × Miyun also significantly deviated from the 3 : 1 ratio (χ^2^= 7.40, *P* = 0.004). No Mendelian segregation was observed for the herbicide-resistant transgene in the hybrids. Visual examination revealed obvious differences in colour and size of both the leaf and the seed among wild soybean, cultivars, and F_1_ and F_2_ plants. The hybrid progeny had various leaf sizes, especially in F_2_ plants (data not shown), as well as various seed colours (yellow, black and green) and different seed sizes, especially in F_3_ seeds, compared with the cultivated and wild soybean **[see** **Supporting Information—Fig. S2****]**.

**Table 3.** Segregation rate of transgene presence in selfing seeds (F_2_) of GM hybrids (F_1_). *P < 0.05, **P < 0.01.

Hybridization	Tested plants	No. of plants with transgene	Percentage of transgene presence	Theoretical segregation rate	*χ*^2^ value
F_2_ [selfing of F_1_ (Miyun × GM AG5601)]	49	28	57	3 : 1	7.40**
F_2_ [selfing of F_1_ (Pinggu × GM AG5601)]	75	47	63	3 : 1	5.44*

### Plant growth

The various plant types showed significantly different vegetative growth periods (*F*_4, 45_ = 23.343, *P* = 0.000 in the greenhouse; *F*_4, 127_ = 10.729, *P* = 0.000 in the field trial) and 100-seed weight (*F*_4, 45_ = 103.218, *P* = 0.000 in the greenhouse; *F*_4, 127_ = 27.316, *P* = 0.000 in the field trial) in both greenhouse and field tests. In addition, the difference among various plant types in the greenhouse was significant for pod setting (*F*_4, 45_ = 2.906, *P* = 0.032) and seed number (*F*_4, 45_ = 4.109, *P* = 0.006), while above-ground biomass was only borderline significant (*F*_4, 45_ = 2.573, *P* = 0.050). For the F_2_ hybrids and wild plants in the field, however, the above-ground biomass significantly differed among various plant types (*F*_4, 127_ = 20.404, *P* = 0.000).

GM F_1_-Miyun had a significantly longer vegetative growth period than that of non-GM F_1_-Miyun (*P* < 0.05, Table 4). However, both F_2_ of GM and non-GM crossing combinations with Miyun wild soybean had similar vegetative growth periods in the field. The vegetative growth period of F1 hybrids was not different from that wild parents except non-GM F1-Miyun that showing earlier flowering (*P* < 0.05), and the vegetative growth period of all GM F_2_ plants was significantly shorter than that of wild soybean in the field (*P* < 0.05).

**Table 4.** Means (±SE) of fitness-related growth characteristics of F_1_ and F_2_ progenies. Different lowercase letters indicate significance at the 0.05 level.

Year	Plant type	Vegetative growth period (days)	Above-ground biomass (g)	Pod number	Seed number	Hundred-seed weight (g)
2010 (greenhouse)	Wild soybean Miyun	105 ± 0.694^b^	29.3 ± 1.18^ab^	163 ± 29^ab^	227 ± 64^b^	0.98 ± 0.036^c^
	GM F_1_-Miyun	104 ± 0.548^b^	32.1 ± 2.63^a^	146 ± 14^b^	153 ± 22^b^	2.98 ± 0.121^b^
	Non-GM F_1_-Miyun	100 ± 0.373^c^	24.2 ± 5.51^b^	159 ± 13^b^	230 ± 21^b^	3.63 ± 0.110^a^
	Wild soybean Pinggu	112 ± 0.553^a^	24.0 ± 3.10^b^	245 ± 15^a^	413 ± 43^a^	0.88 ± 0.018^c^
	GM F_1_-Pinggu	108 ± 1.580^ab^	27.7 ± 1.76^ab^	158 ± 16^b^	190 ± 33^b^	2.82 ± 0.242^b^
	Non-GM F_1_-Pinggu	—	—	—	—	—
2011 (field)	Wild soybean Miyun	93 ± 1.033^a^	93.5 ± 16.01^c^	797 ± 123^a^	1130 ± 269^a^	0.94 ± 0.039^b^
	GM F_2_-Miyun	86 ± 0.756^b^	261.0 ± 25.11^b^	941 ± 123^a^	1184 ± 207^a^	2.99 ± 0.193^a^
	Non-GM F_2_-Miyun	86 ± 0.780^b^	246.1 ± 19.27^b^	1002 ± 96^a^	1490 ± 187^a^	3.06 ± 0.120^a^
	Wild soybean Pinggu	93 ± 1.085^a^	133.7 ± 26.84^c^	1095 ± 168^a^	1572 ± 408^a^	1.09 ± 0.207^b^
	GM F_2_-Pinggu	86 ± 0.747^b^	417.1 ± 25.02^a^	1394 ± 160^a^	1576 ± 209^a^	3.06 ± 0.135^a^
	Non-GM F_2_-Pinggu	—	—	—	—	—

The dry weight of the above-ground biomass of GM F_1_ hybrids was significantly higher than that of non-GM hybrids (*P*< 0.05, Table 4), while GM F_2_ plants formed with the wild parent Miyun had slightly higher above-ground biomass than their non-GM counterparts, but the difference was not significant. In addition, both GM and non-GM F_2_ had a significantly higher above-ground biomass (*P*< 0.05) than wild soybean in the field.

Wild soybean Pinggu, the wild parent, set a significantly larger amount of pods and more seeds than its F_1_ GM hybrids in the greenhouse (*P*< 0.05, Table 4). However, there was no significant difference in pod number of F_1_ or F_2_ between GM and non-GM plants. In the field trial, there was no significant difference between F_2_ progeny and all wild soybean parents for seed production. Although the GM F_2_ hybrids produced fewer seeds than the non-GM ones, the difference was not significant in the field.

Both GM and non-GM hybrids produced significantly higher 100-seed weight than the wild soybean parent in the greenhouse (*P* < 0.05, Table 4) for the hybrids formed with wild soybean Miyun, and the 100-seed weight produced by the GM hybrid F_1_ was significantly lower than that of the non-GM hybrid (*P* < 0.05). The GM hybrids that formed with wild soybean Pinggu also produced higher 100-seed weight than their wild parent (*P* < 0.05). In addition, the 100-seed weight of the seeds set by F_2_ progeny was significantly higher than that of the wild soybean parent of both geographical populations in the field (*P* < 0.05). The non-GM F_2_-Miyun set virtually the same 100-seed weight as the GM F_2_-Miyun.

The results showed that there was no difference in plant growth performance between GM and non-GM F_2_ hybrids in the field, while GM hybrid F_1_ plants had a significantly longer period of vegetative growth, higher biomass and lower seed weight than the non-GM F_1_ plants in the greenhouse test. The 100-seed weight of both F_1_ and F_2_ hybrids was significantly higher than that of wild soybean.

## Discussion

Although the probability of natural hybridization between wild soybeans and GM soybeans may be low under field conditions, a few studies have suggested that the persistence of foreign genes introgressing into wild populations depended on the survival and fecundity of hybrids and the fitness of the introgressed genes (Di *et al*. 2009; Shivrain *et al*. 2009). Plant performance of hybrids predicts the fitness of the introgressed genes after hybridization. However, no researchers have reported on the performance of hybrids between wild soybeans and GM soybeans in the field. Component performance may be used to predict the fate of GM hybrids in the field. If the transgene has an adverse effect, it could reduce the persistence of the plants in the field (Di *et al*. 2009; Song *et al*. 2011; Wang *et al*. 2014). However, no difference in plant performance was found here between GM and non-GM hybrids in the field trial, which suggests that there is no adverse impact of the herbicide-resistant gene. Although seed germination and survival should also be investigated in further study, the result reported here is consistent with other reports showing that the EPSPS gene did not alter the developmental and agronomic traits of soybean (e.g. Wu *et al*. 2007; Lü *et al*. 2009).

Soybean is self-compatible with a low outcrossing rate. The failure of pollination in the Pinggu wild population might be due to potential genetic isolation or sexual incompatibility. However, many other factors may also affect the success of hybridization, such as pollen viability and pistil receptivity (Huang *et al*. 2004). Possible explanations for the absence of the transgene in the seed set of the wild female plant are that the sterilization in wild soybean was incomplete or that self-fertilization took place before hybridization in the wild soybean.

In order to assess the performance of a hybrid, it is crucial to select appropriate indexes that properly reflect the competition and reproductive potential of that variant (Di *et al*. 2009; Song *et al*. 2011). In this study, in the absence of herbicide pressure, some performance-related characteristics of GM hybrids were determined in both the greenhouse and the field. The GM soybean AG5601 and non-GM SKN500 were provided by Monsanto Company as paired lines for this study. The growth of these two lines in the field was similar (Wu *et al*. 2007). The difference between GM F_1_ and related non-GM F_1_ of the same maternal plant was thus assumed to be caused by the insertion of transgenes. In addition, there seemed to be a trade-off between biomass and seed production in the F_1_ progeny of soybean and wild soybean Miyun, where high reproductive growth resulted in reduced vegetative plant size in non-GM F_1_-Miyun hybrid plants due to the cost of reproduction (Obeso 2002).

The results indicated that although F_1_ hybrid progeny obtained by crossing between wild soybean and GM soybeans had lower pod setting percentages and seed number than wild soybean parents, F_2_ progeny had shown higher performance in the field. Some genetic variations existed between GM cultivated plants and wild relatives, and some features (e.g. rapid growth and early flowering) may enhance hybrids fitness (Mercer *et al*. 2007; Wei *et al*. 2012). In our research, 100-seed weight and above-ground biomass in GM and non-GM plants of F_1_ and F_2_ were higher than those values in wild soybeans. It is not surprising that the traits introgressed from a cultivated paternal parent would enhance plant performance of the hybrid (Mercer *et al*. 2007). The increased growth of hybrids compared with wild plants in our study might be due to the paternal effect and/or the presence of heterosis. Although low replicates of maternal plants during hybridization could limit the genetic diversity of hybrid progeny from drawing a much broader conclusion, it might be able to reduce the variation in plant performance during comparison in this study.

Herbicide resistance has a selective advantage at any level of herbicide application in farmland. This advantage could increase the persistence ability of this transgene in farmland, where herbicides are routinely applied (Warwick *et al*. 2008). In addition, the herbicide-resistant transgene will also likely be retained even in the absence of selective pressure (herbicide application) as long as it does not have a significant adverse effect. Scientists have reported that GM feral oilseed rape populations have established in areas where there is no herbicide application at all and that they can persist outside cultivated areas (Snow *et al*. 1999; Warwick *et al*. 2008; Schafer *et al*. 2011). Similar results are also found in other plants (Zhang *et al*. 2003; Guadagnuolo *et al*. 2006). The assessment of transgene flow from a glyphosate-resistant transgenic soybean AG5601 (the same type that we used) to conventional soybeans in China indicated that transgenic soybean AG5601 may result in a risk of gene flow via pollination and transgene escape to compatible relatives (Huang *et al*. 2014). The absence of an adverse effect in GM hybrids could lead to the persistence of the transgene in wild plants (Di *et al*. 2009; Liu *et al.* 2012). Wang *et al*. (2014) suggested that over-expression of the herbicide resistance (*epsps*) gene could result in fitness benefits in weedy rice relatives following transgene introgression. This suggestion is consistent with our result, which indicated that the herbicide-resistant gene might not adversely affect the growth of introgressed wild soybeans and therefore could be expected to persist in nature.

## Conclusions

Studies have demonstrated that gene flow between cultivated soybean and wild soybean has actually occurred due to frequent visits by pollinators, such as honeybees and carpenter bees (Nakayama and Yamaguchi 2002; Wang *et al*. 2010). There were few signs of decreasing viability and vigour in the F_1_ and F_2_ hybrids in our study, and it is plausible that they showed active vegetative growth due to heterosis. These advantages could eventually cause potential transgene escape from GM soybeans into wild soybean populations and could allow the transgene to be passed down to future generations. As GM soybeans are increasingly cultivated, especially in areas that harbour populations of wild soybeans, the ecological risk and consequence of gene flow from GM soybeans with traits of selective advantage (such as resistance to herbicides, insect pests or other biotic and abiotic factors) deserves special attention. The fate of such resistance transgenes and their ecological effects should be assessed and evaluated before GM soybeans are commercially released in order to assure the maximum benefit of GM crops with minimum risk to the environment.

## Sources of Funding

This work is supported by the China National Special Transgenic Projects ‘Monitoring and controlling technology for ecological risks in nature (2011ZX08012-005)’ and ‘Environmental evaluation techniques for genetically modified soybean, maize and wheat (2008ZX08011-003)’, which are managed by the Ministry of Agriculture of China. Z.-J.G. acknowledges the financial support from the National Natural Science Foundation of China (no. 31200422), China Postdoctoral Science Foundation (nos 2012M520455 and 2013T60193) and Yuncheng University Doctor Scientific research project (YQ-2014022).

## Contributions by the Authors

W.W. and D.-M.K. conceived and designed the experiments; P.-F.Z. and Z.-J.G. performed the experiments; Z.-J.G., W.W. and X.-C.M. analysed the data; W.W. and B.L. contributed reagents/analysis tools and Z.-J.G., W.W. and D.-M.K. wrote the paper.

## Conflict of Interest Statement

None declared.

## Supporting Information

The following additional information is available in the online version of this article –

**Figure S1****.** Polymerase chain reaction identification of GM hybrids and SSR detection of non-GM hybrids.

**Figure S2****.** Seed sizes of wild soybean, cultivated soybean and hybrids (F_1_, F_2_ and F_3_).

Additional Information
