# Supplementary material for: Performance of hybrid progeny formed between genetically modified herbicide-tolerant soybean and its wild ancestor
Source: AoB Plants. 2015 Oct 27;7:plv121. doi: 10.1093/aobpla/plv121 (PMC4670487; doi:10.1093/aobpla/plv121)
Supplement: Additional Information [file supp_7_plv121_index.html]

Performance of hybrid progeny formed between genetically modified herbicide-tolerant soybean and its wild ancestor — Performance of hybrid progeny formed between genetically modified herbicide-tolerant soybean and its wild ancestor — Additional Information 

# Performance of hybrid progeny formed between genetically modified herbicide-tolerant soybean and its wild ancestor

## Additional Information

Additional Information

- Additional Information - Doc file
