# Supplementary material for: Performance of hybrid progeny formed between genetically modified herbicide-tolerant soybean and its wild ancestor
Source: AoB Plants. 2015 Oct 27;7:plv121. doi: 10.1093/aobpla/plv121 (PMC4670487; doi:10.1093/aobpla/plv121)
Supplement: Additional Information [file supp_plv121_plv121supp.doc]

Supporting Information

**Figure S1**


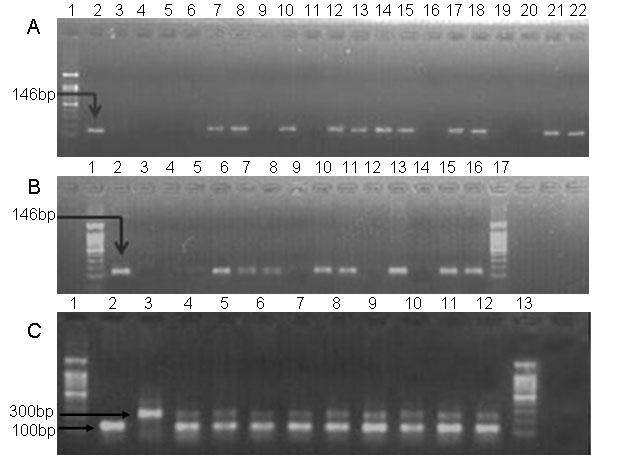


**Figure S1** PCR identification of GM hybrids and SSR detection of non-GM hybrids. **A**. PCR identification of GM F_1_ hybrids. 1, Molecular marker; 2, GM soybean; 3, wild soybean; 4, blank control; 5-22, F_1_ hybrids. The arrow indicated EPSPS gene fragment of 146 bp. **B**. PCR identification of GM F_2_ plants. 1 and 17, Molecular marker; 2, GM soybean; 3, wild soybean; 4, blank control; 5-16, F_2_ plants. The arrow indicated EPSPS gene fragment of 146 bp. **C**. SSR detection of non-GM F_1_ hybrids. 1 and 13, Molecular marker; 2, Miyun wild soybean (female parent); 3, non-GM soybean SKN500 (male parent); 4-12, F_1_ hybrids. The arrows indicated male (300bp) and female (100bp) parent-speciﬁc markers.

**Figure S2**


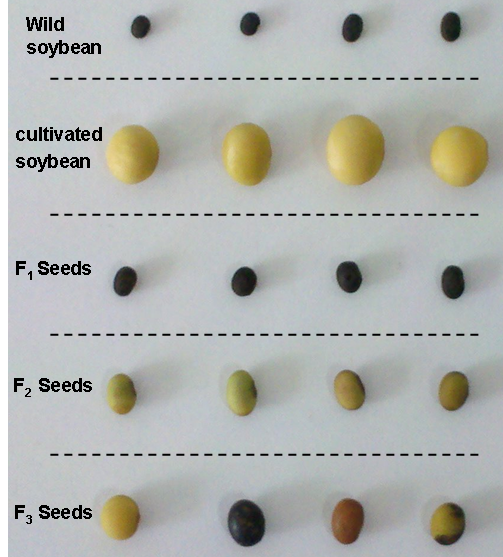


Figure S2 Seed size of s different plant types: wild soybean, cultivated soybean, F_1_ seeds, F_2_ seeds (harvested in the greenhouse) and F_3_ seeds (harvested in the field).
